# Sex-specific effects of the in ovo environment on early-life phenotypes in eiders

**DOI:** 10.1007/s00442-019-04569-9

**Published:** 2019-11-30

**Authors:** Markus Öst, Kristina Noreikiene, Frederic Angelier, Kim Jaatinen

**Affiliations:** 1 Environmental and Marine Biology, Åbo Akademi University, Turku, Finland; 2 Novia University of Applied Sciences, Ekenäs, Finland; 3 Chair of Aquaculture, Institute of Veterinary Medicine and Animal Sciences, Estonian University of Life Sciences, Kreutzwaldi tn. 46, Tartu, Estonia; 4 Centre d’Etudes Biologiques de Chizé, CNRS, La Rochelle Université, UMR 7372, Villiers en Bois, France; 5 Nature and Game Management Trust Finland, Degerby, Finland

**Keywords:** Early environment, Feather corticosterone, Prenatal growth, *Somateria mollissima*, Telomere length

## Abstract

Maternal effects affect offspring phenotype and fitness. However, the roles of offspring sex-specific sensitivity to maternal glucocorticoids and sex-biased maternal investment remain unclear. It is also uncertain whether telomere length (a marker associated with lifespan) depends on early growth in a sex-specific manner. We assessed whether maternal traits including corticosterone (CORT; the main avian glucocorticoid) and in ovo growth rate are sex-specifically related to offspring CORT exposure, relative telomere length (RTL) and body condition in eiders (*Somateria mollissima*). We measured feather CORT (fCORT), RTL and body condition of newly hatched ducklings, and growth rate in ovo was expressed as tarsus length at hatching per incubation duration. Maternal traits included baseline plasma CORT, RTL, body condition and breeding experience. We found that fCORT was negatively associated with growth rate in daughters, while it showed a positive association in sons. Lower offspring fCORT was associated with higher maternal baseline plasma CORT, and fCORT was higher in larger clutches and in those hatching later. The RTL of daughters was negatively associated with maternal RTL, whereas that of males was nearly independent of maternal RTL. Higher fCORT in ovo was associated with longer RTL at hatching in both sexes. Duckling body condition was mainly explained by egg weight, and sons had a slightly lower body condition. Our correlational results suggest that maternal effects may have heterogeneous and even diametrically opposed effects between the sexes during early development. Our findings also challenge the view that prenatal CORT exposure is invariably associated with shorter telomeres.

## Introduction

Early-life exposure to glucocorticoids may have a profound effect on offspring phenotype, either preparing the offspring for their postnatal environment or decreasing their fitness value (Haussmann et al. 2012; Herborn et al. 2014; Monaghan and Haussmann 2015). Together with maternal attributes such as breeding experience, body condition and reproductive investment, maternal glucocorticoids are thought to shape the early-life environment of offspring (Love et al. 2005; Monaghan 2008). An intriguing but as yet little explored possibility is that mothers are capable of shaping the offspring phenotype in a sex-specific manner (Young and Badyaev 2004; Love et al. 2005; Badyaev et al. 2006). In addition, developing embryos, though conventionally viewed as merely passive receivers of maternal endocrine signals, may in fact be capable of metabolizing glucocorticoids, thus modifying maternal effects (Reed and Clark 2011; Vassallo et al. 2014). Further, the impact of glucocorticoids on the developing embryo can differ depending on its sex (Butkevich et al. 2009; Jimeno et al. 2017). Such sex-specific effects of maternal glucocorticoids may arise due to differential regulation of the hypothalamic–pituitary axis between the sexes (Schmidt et al. 2014). Therefore, postnatal phenotypic differences between the sexes may be the product of a complex interplay between sex-specific maternal deposition of substances into eggs, the ability of embryos to modulate the effects of these substances, and interactions between maternal deposition and embryonic modulation (Vassallo et al. 2014; Groothuis et al. 2019).

Telomeres, the protective ends of chromosomes, may represent a potential mechanism by which variation in early-life glucocorticoid exposure may translate into differential survival prospects (Angelier et al. 2018; Gil et al. 2019). Exposure to glucocorticoids during early-life can severely reduce telomere length (Haussmann et al. 2012; Herborn et al. 2014) and thereby impact survival prospects (Heidinger et al. 2012; Barrett et al. 2013). However, there are also sex differences in lifespan, which may be mirrored in sex-dependent variation in telomere dynamics (Barrett and Richardson 2011; Parolini et al. 2015; Noguera et al. 2015). Such differences may arise due to an unguarded sex chromosome in the heterogametic sex (Barrett and Richardson 2011). Sexes also differ in their susceptibility to glucocorticoids during the developmental stages (Jimeno et al. 2017). Furthermore, resources allocated to growth may be at the expense of resources allocated to somatic maintenance and thereby longevity. Males and females can differ in growth trajectories (Parolini et al. 2015) and in the effects of prenatal glucocorticoid exposure on growth (Tissier et al. 2014). These differences may translate into different resource investment priorities between growth and self-maintenance, ultimately leading to significant differences in developmental telomere attrition between the sexes (Barrett and Richardson 2011). However, it remains largely unknown, both experimentally and in the field, how maternal effects and specifically prenatal exposure to glucocorticoids are linked to sex-specific differences in telomere length (but see Haussmann et al. 2012; Gil et al. 2019).

Cellular stress damage and telomere shortening may be particularly intense during early development, with important consequences for life expectancy and future performance (Heidinger et al. 2012; Monaghan and Ozanne 2018). Despite this importance, non-destructive quantification of glucocorticoid exposure during fetal development is challenging (von Engelhardt and Groothuis 2005). In birds, corticosterone (CORT; the main glucocorticoid in birds) deposited into structures of feather keratin gives a pooled measure of the fluctuations in baseline CORT levels, as well as the frequency, magnitude and duration of stress-induced CORT elevations experienced by the bird over the period of feather growth (Bortolotti et al. 2008; Romero and Fairhurst 2016). Consequently, feather CORT (fCORT) measured at hatching represents a non-destructive way of measuring glucocorticoid exposure in ovo. An additional benefit is that fCORT measured at hatching avoids direct competition-induced stress among nestlings, which may demonstrably induce telomere shortening (e.g., Cram et al. 2017).

Here, we examine the influence of prenatal growth and physiological maternal effects (body condition, breeding experience, CORT level and telomere length) on offspring prenatal CORT exposure (fCORT), body condition and telomere length, and whether any such effects may be sex specific. We also investigate whether CORT exposure while in ovo (measured as fCORT) may be correlated with telomere length and body condition at hatching. To this end, we studied a wild population of eiders (*Somateria mollissima*). This long-lived and precocial species produces a relatively small clutch of large eggs, making it an ideal study model for investigating maternal effects on offspring phenotype. Importantly, there is no significant sexual size dimorphism at hatching in this species (Lehikoinen et al. 2008), and therefore, potential sex differences in our variables of interests will not be mere by-products of size dimorphism.

## Materials and methods

### Field data collection

Incubating females were trapped on their nests using hand nets at Tvärminne (59° 50′ N, 23° 15′ E), southwestern Finland, in 2013. Upon capture, body weight, radius–ulna length, clutch size, clutch weight, and incubation stage, using egg floatation, were recorded. The estimated incubation stage does not statistically differ from the real incubation stage based on direct observation (Kilpi and Lindström 1997). Blood samples (< 1 ml) were taken within 3 min (mean ± SD = 145 ± 21 s, *N* = 199) of female capture, and immediately stored on ice in a cool box and transported to the laboratory within 2–4 h. Blood plasma was separated from blood cells by centrifugation and both components were stored in − 80 °C until further analyses. Minimum years of maternal experience (hereafter, breeding experience) was calculated as the number of years since the bird was first trapped; chronological age could not be determined because females are not ringed as ducklings. This is a reasonably good proxy for female age in this population annually ringed since 1990 due to the high breeding philopatry and because the majority of breeding females are captured in each year (Öst and Steele 2010; Jaatinen and Öst 2011). Nevertheless, age can only be estimated and not accurately determined in our study, especially because there is some variation in the age at first breeding (typically 3 years, range 2–5 years; Hario and Rintala 2009). A female’s body condition was estimated as body weight at hatching of her clutch corrected for structural size, i.e., the standardized residuals from a linear regression of log-transformed projected weight at hatching on log-transformed radius–ulna length (Öst et al. 2008a; Öst and Steele 2010). Projected weight at hatching was obtained by subtracting an estimate of the expected body weight loss during the remaining incubation time from measured incubation body weight. Females were weighed once, but as they abstain from feeding during incubation and were captured at different times in their incubation, we can derive an estimate of mean weight loss rate during incubation as the slope of the regression of log-transformed body mass (response variable) on log-transformed incubation time and projected hatching date (Öst et al. 2008a).

We estimated relative reproductive investment as standardized residual mean egg weight. Each clutch was weighed to the nearest 1 g and mean egg weight was calculated. To correct for eggs becoming lighter with progressing incubation, standardized residual mean egg weight was represented by the standardized residuals from a linear regression of log-transformed mean egg weight on log-transformed incubation stage at capture. Clutches exceeding 7, the maximum laid by one female (Waldeck et al. 2004), were excluded from further analyses due to the presence of parasitic eggs. Although clutches smaller than the above threshold may also contain some parasitic eggs, this frequency is low in our study population (ca. 6% parasitically laid eggs; Waldeck et al. 2004).

Ducklings leave the nest within 24 h of hatching, and thus, consecutive nest visits were planned to coincide with hatching date estimated by egg floatation. Ducklings (*N* = 304/88 broods) were captured in the nest, weighed, and their tarsus length recorded. Growth rate in ovo was expressed as tarsus length divided by incubation duration. The duration of the incubation period was determined as the time difference (in days) between the real hatch date observed during nest visits and the estimated date of incubation onset determined by egg floatation at female capture (Seltmann et al. 2012). A small blood sample (< 50 µl) and 1–2 tail feathers were collected. Duckling blood samples were immediately stored on ice in a cool box, transported to the laboratory within 2–4 h, and stored frozen in − 80 °C until further analyses. Duckling body condition was given by body weight corrected for structural size to separate aspects of body weight that are due to structural size from aspects that reflect energy reserves (‘condition’). Body condition was, therefore, estimated as the standardized residuals from a linear regression of log-transformed body weight on log-transformed tarsus length.

### Molecular sex determination

Duckling sex was determined by molecular sexing which is based on the amplification of a part of the gene for the chromo-helicase DNA binding protein and yields different-sized amplicons in female and male eiders (Fridolfsson and Ellegren 1999). Of the totally 285 sexed ducklings, 141 (49.5%) were male, not significantly deviating from an even sex ratio (binomial test: *P* = 0.91).

### Telomere assay

We measured relative telomere length (RTL) in red blood cells using real-time quantitative PCR (qPCR) (Cawthon 2002; Criscuolo et al. 2009), using an assay that we have previously validated for use in female eiders (Noreikiene et al. 2017). Genomic DNA was extracted using the salt-extraction method (Aljanabi and Martinez 1997) and DNA quality was controlled spectrophotometrically and with agarose gel electrophoresis. The telomere assay included amplification of a standard gene (glyceraldehyde 3-phosphate dehydrogenase gene; *gapdh*) using primers developed for chicken *gapdh* Fw.: (5′-TCCTGTGACTTCAATGGTGA-3′) and *gapdh* Rev.: (5′-AAACAAGCTTGACGAAATGG-3′) and telomeric repeats using universal primers (Cawthon 2002). Both telomere and *gapdh* reactions were carried out in triplicate on the same plate in BIO-RAD X1000 real-time thermal cyclers (BIO-RAD) using iQ™ SYBR^®^ Green qPCR mix (BIO-RAD). Every plate also included serial doubling dilutions of a standard sample, which was later used to construct standard curves. Further details on the application of the telomere assay to the current study population can be found elsewhere (Noreikiene et al. 2017). The mean qPCR efficiencies as determined by the standard curves for telomere and *gapdh* reactions fell within the acceptable range of 85–115% (Bize et al. 2009). The intra-plate CVs for telomeres and *gapdh* reactions were 2% and 1.5%, respectively. Inter-plate CVs were 5% for telomeres and 3% for *gapdh*. A relative TL (RTL) was calculated by taking qPCR efficiencies into consideration (Pfaffl 2001).

### Corticosterone assays

CORT in female blood plasma and in feathers of ducklings was analyzed using radioimmunoassay. An important note to make here is that female baseline CORT levels in blood measured at different incubation stages may not accurately reflect corticosterone deposited in eggs at the time of laying. Nevertheless, baseline blood corticosterone levels in female eiders do not vary depending on incubation stage at capture, and show individual consistency even between different breeding seasons (Jaatinen et al. 2013). It is, therefore, conceivable that the maternal baseline plasma CORT concentrations measured here are fairly closely correlated with actual maternal corticosterone deposited in eggs. Adult female plasma CORT was measured using a double antibody kit (ImmuChemTMMPBiomedicals, Orangeburg, NY). A validation of the plasma CORT RIA kit for female eiders, including the extraction method, is given by Nilsson (2004). Duckling feather corticosterone (fCORT) was analyzed according to Bortolotti et al. (2008) with slight modifications. For each duckling, an unwashed feather was measured (length) with a caliper to the nearest 0.1 mm. Then, 10 ml of methanol (HPLC grade) was added to each feather to extract CORT. The feathers were placed in a sonicating water bath at room temperature for 30 min, followed by incubation at 50 °C overnight in a shaking water bath. The methanol was then separated from feather material by filtration, using filtered syringes. The methanol extract was then placed in a water bath (50 °C) and methanol was evaporated in a fume hood. The extract was reconstituted in a small volume of phosphate buffer system (PBS; 0.05 m, pH 7.6). All extracts were subsequently analyzed by radio-immunoassay as previously described (Meillère et al. 2016). All samples were run in 6 assays and the intra- and inter-assay CVs were, respectively, 9.34% and 11.65%. Samples were randomly distributed in the assays. Duckling fCORT incorporates all CORT exposure experienced during development (ca. 26 days) and up to 24 h post-hatch.

### Statistical analyses

We investigated the degree to which a common in ovo environment (maternal effects) and genetic background affected duckling fCORT, RTL and body condition by calculating the within-brood repeatability for these variables. Within-brood repeatability is given by the intraclass correlation coefficient, representing the fraction of total phenotypic variance that can be attributed to variation among ducklings in the same brood. High within-brood repeatability indicates that the common environment and/or genetic effects are important determinants of duckling fCORT, RTL and body condition. Within-brood repeatability was calculated using the rptR package (Stoffel et al. 2017).

To analyze whether duckling fCORT, RTL and body condition are associated with in ovo growth, maternal traits and CORT levels in a sex-specific manner, we constructed three linear mixed models (LMMs) based on restricted maximum likelihood (REML) parameter estimation. In these LMMs, the response variables duckling fCORT, RTL and body condition, respectively, were explained by duckling sex and growth rate, maternal blood plasma CORT, and maternal RTL, breeding experience and body condition. The two-way interactions between offspring sex and maternal traits, as well as between offspring sex and growth rate, were included in all three models. Standardized residual mean egg weight corrected for incubation stage was included in all models to control for effects on offspring phenotype due to differences in maternal reproductive investment, which may influence, e.g., offspring telomere lengths (McLennan et al. 2018). Also clutch size and hatch date may influence offspring telomere dynamics (Heidinger et al. 2016) and were, therefore, included as covariates. Time to maternal blood sample was included as a technical covariate. We also investigated the potential impact of CORT in ovo on RTL and body condition at hatching, by including fCORT as a predictor in the analysis of duckling RTL and body condition. Mother identity was included as a random factor in all models to account for interdependence of observations from the same broods. Duckling telomere lengths were mean-centered within plates (mean ± SE = 30.6 ± 5.2 ducklings per plate, *N* = 8 plates/245 ducklings) to control for significant but non-informative differences in mean RTL between plates. Mean-centring allowed us to adjust for plate-related bias without affecting relative differences in RTL between individuals. This approach was further justified by the reasonable large sample size per plate. Duckling RTL and fCORT levels were log-transformed in order for the residuals of all models to adhere to the assumption of normality.

We identified final models in which all covariates were statistically significant (*α* = 0.05) using stepwise model reduction, which is considered a conservative yet powerful model selection strategy (Murtaugh 2009). Briefly, we compared model deviance with and without each fixed effect using likelihood ratio tests until only significant terms remained. All analyses were conducted using the statistical software R 3.4.3 (R Core Team 2017). Due to missing data on some variables, the final sample size was 181 ducklings [89 males (49.2%)] and 58 adult females in the analyses of duckling fCORT and body condition, and 156 ducklings [77 males (49.4%)] and 56 adult females in the analysis of duckling RTL.

## Results

### Duckling fCORT

**Fig. 1:**
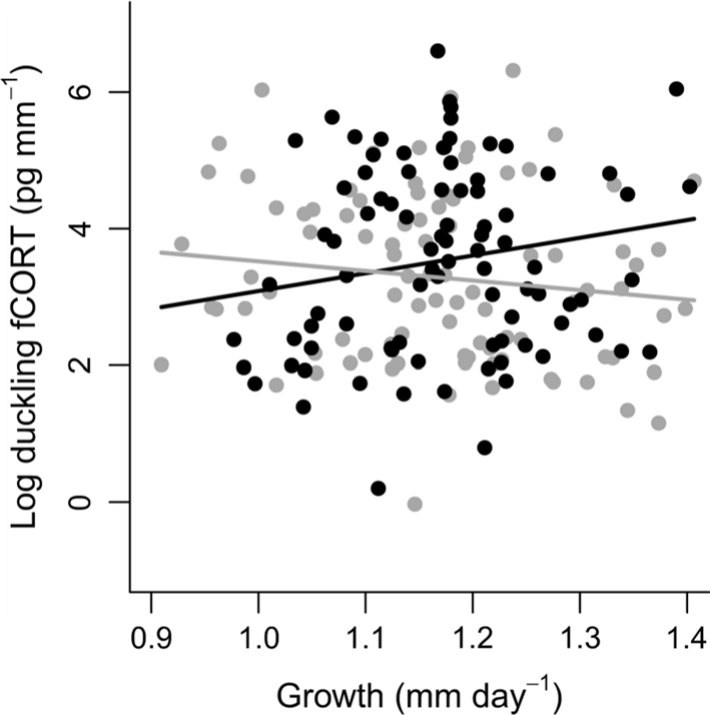
Duckling feather corticosterone (fCORT) at hatching as a function of growth in ovo (tarsus length at hatching divided by incubation duration) and offspring sex. Feather corticosterone of female offspring (gray line and circles) decreases with increasing growth rate, while that of male offspring (black line and circles) is positively associated with growth

**Fig. 2:**
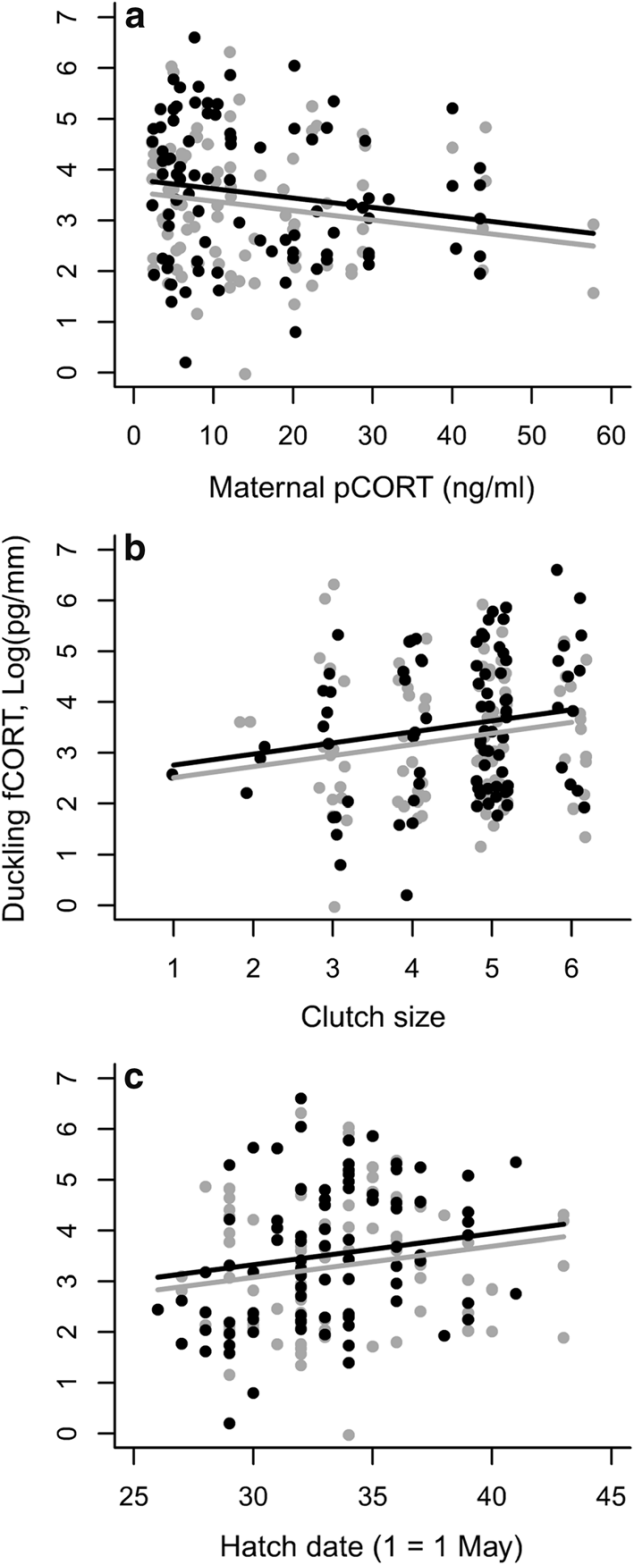
Duckling feather corticosterone (fCORT) at hatching as a function of **a** maternal baseline plasma corticosterone (pCORT), **b** clutch size, and **c** hatching date. Data on female (gray line and circles) and male offspring (black line and circles) are separated by sex for illustrative purposes only, and values for clutch size are jittered for visual clarity

Duckling fCORT showed relatively low but significant within-brood repeatability (*r* = 0.25, 95% CI 0.13–0.37, *P* < 0.001), which suggests that the fCORT levels of ducklings from the same brood resemble each other. Offspring sex and growth rate in ovo interactively explained variation in offspring fCORT (Table 1): lower fCORT of daughters was associated with increasing growth rate, while fCORT levels and growth were positively correlated in sons (Fig. 1). Lower offspring fCORT was also associated with higher maternal baseline plasma CORT concentrations (Fig. 2a), and duckling fCORT was higher in larger clutches (Fig. 2b) and in clutches hatching later (Fig. 2c). Duckling fCORT was not related to maternal RTL, body condition, breeding experience or standardized residual egg weight (Table 1).

**Table 1:** Final linear mixed effect model (in bold) and model selection of variables and interactions explaining duckling feather corticosterone (fCORT)

Dependent variable	Predictor variables	Estimate ± SE	*t*	*P*
Log(duckling fCORT) (pg/mm)	Sex (male)	**− 4.44 ± 2.16**	**− 2.06**	**0.04**
	Maternal plasma CORT (ng/ml)	**− 0.018 ± 0.009**	**− 2.06**	**0.04**
	Clutch size	**0.22 ± 0.10**	**2.13**	**0.04**
	Hatch date	**0.06 ± 0.03**	**2.05**	**0.045**
	Growth (tarsus length incubation duration^−1^)	**− 1.40 ± 1.24**	**− 1.13**	**0.26**
	Sex (male) × growth	**4.00 ± 1.83**	**2.18**	**0.03**
	Maternal body condition	0.26 ± 0.15	1.65	0.10
	Sex × maternal RTL	**− **0.28 ± 0.18	**− **1.61	0.11
	Sex × maternal plasma CORT	**− **0.014 ± 0.02	**− **0.89	0.38
	Breeding experience (years)	**− **0.023 ± 0.03	**− **0.84	0.41
	Sex × maternal body condition	**− **0.20 ± 0.28	**− **0.72	0.47
	Sex × breeding experience	0.044 ± 0.05	0.83	0.41
	Time to maternal blood sample (s)	0.00039 ± 0.0062	0.062	0.95
	Standardized residual egg weight	0.0073 ± 0.12	0.062	0.95

Given are the parameter estimates, standard errors (SE), *t* values and the *P* values; the model included maternal ID as a random effect (*N* = 181 ducklings and 58 females)

### Duckling RTL

**Fig. 3:**
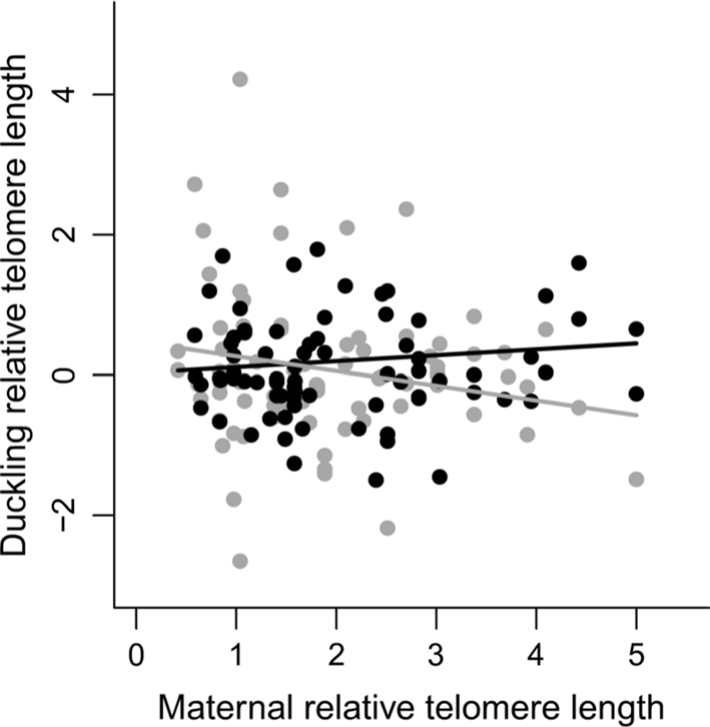
Duckling relative telomere length at hatching (log-transformed and mean-centered within plates, see “Statistical analyses”) as a function of maternal relative telomere length. The relative telomere length of daughters (gray line and circles) shows a negative relationship with maternal relative telomere length, whereas that of sons (black line and circles) is essentially independent of maternal relative telomere length

**Fig. 4:**
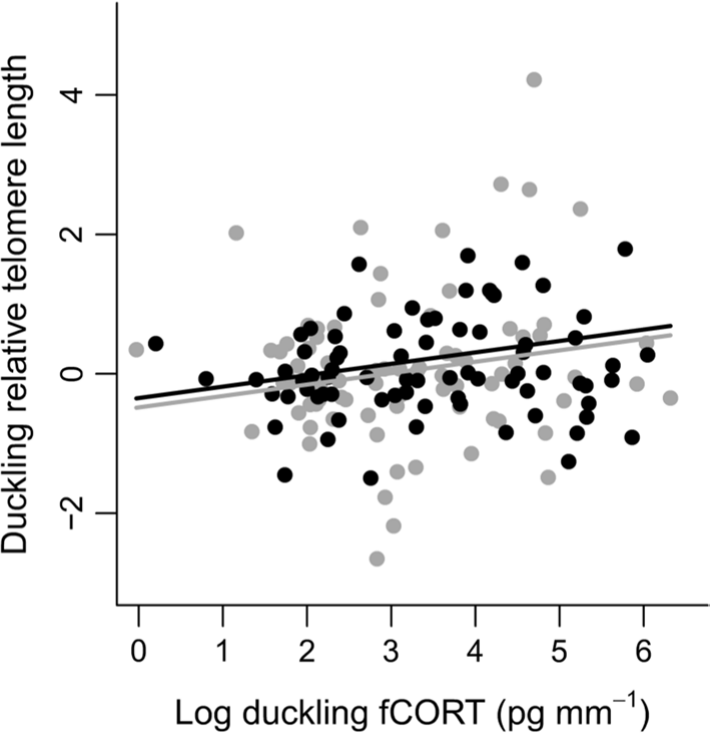
Relative telomere length of ducklings as a function of their feather corticosterone (fCORT) level at hatching. Data on female (gray line and circles) and male offspring (black line and circles) are separated by sex for illustrative purposes

Duckling RTL showed low but significant within-brood repeatability (*r* = 0.15, 95% CI 0.01–0.29, *P* = 0.01), indicating that the telomere lengths of brood-mates were correlated. Maternal RTL had a sex-specific association with offspring RTL (Table 2). The RTL of daughters was negatively associated with maternal RTL, whereas that of males was nearly independent of maternal RTL (Fig. 3). Higher fCORT in ovo was associated with longer RTL at hatching in both sexes (Fig. 4). No other variables or interactions were found to be significantly related to duckling RTL (Table 2).

**Table 2:** Final linear mixed effect model (in bold) and model selection of variables and interactions explaining duckling relative telomere length (RTL)

Dependent variable	Predictor variables	Estimate ± SE	*t*	*P*
Duckling RTL	Sex (male)	**− 0.45 ± 0.33**	**− 1.35**	**0.18**
	Log(duckling fCORT) (pg/mm)	**0.16 ± 0.062**	**2.65**	**0.009**
	Maternal RTL	**− 0.21 ± 0.11**	**− 1.85**	**0.07**
	Sex (male) × maternal RTL	**0.29 ± 0.15**	**2.01**	**0.046**
	Standardized residual egg weight	**− **0.10 ± 0.097	**− **1.05	0.30
	Growth (tarsus length incubation duration^−1^)	0.65 ± 0.83	0.79	0.44
	Breeding experience (years)	0.0036 ± 0.024	0.15	0.88
	Sex × breeding experience	0.029 ± 0.043	0.66	0.51
	Maternal plasma CORT (ng/ml)	**− **0.0017 ± 0.0080	**− **0.22	0.83
	Sex × maternal plasma CORT	**− **0.011 ± 0.015	**− **0.75	0.46
	Time to maternal blood sample (s)	**− **0.0034 ± 0.0056	**− **0.61	0.55
	Clutch size	**− **0.052 ± 0.11	**− **0.48	0.63
	Growth × sex	**− **0.71 ± 1.59	**− **0.45	0.66
	Maternal body condition	**− **0.041 ± 0.16	**− **0.26	0.80
	Sex × log(duckling fCORT)	**− **0.047 ± 0.13	**− **0.36	0.72
	Hatch date	0.005 ± 0.029	0.18	0.86
	Sex × maternal body condition	**− **0.067 ± 0.29	**− **0.23	0.82

RTL values were log-transformed and mean-centered within plates (see “Statistical analyses”). Given are the parameter estimates, standard errors (SE), *t* values and the *P* values; the model included maternal ID as a random effect (*N* = 156 ducklings and 56 females)

### Duckling body condition

**Fig. 5:**
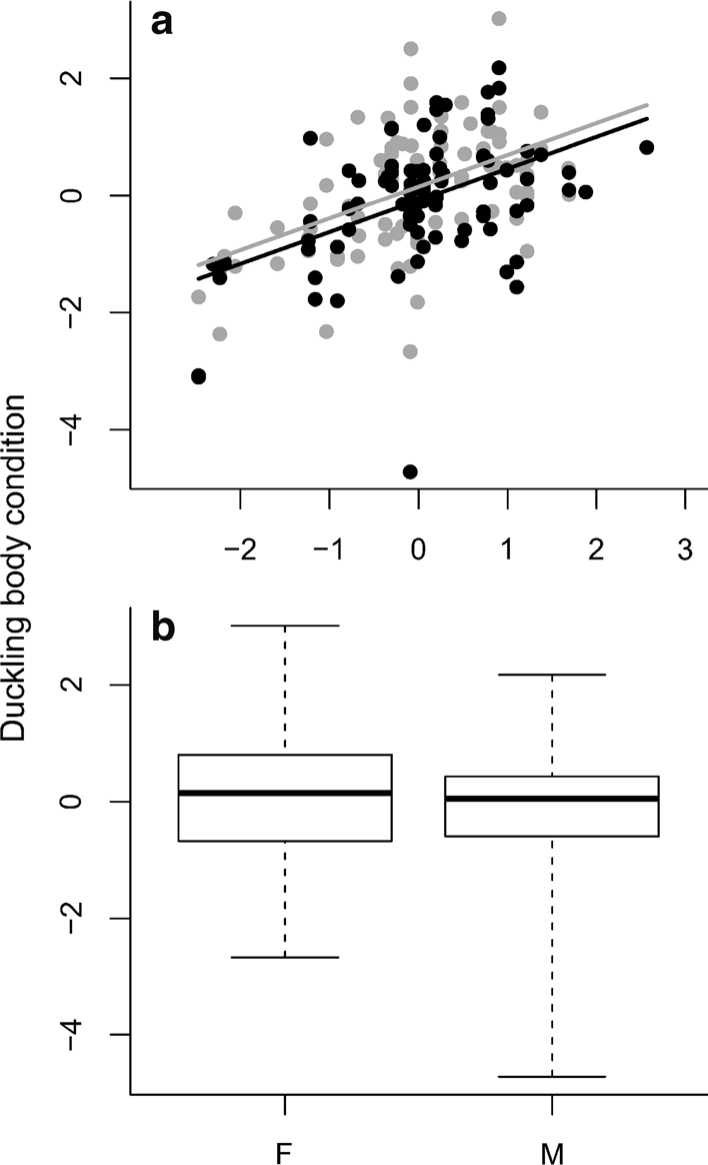
Duckling body condition at hatching (for definition, see text) in relation to **a** standardized residual egg weight (for definition, see text) and **b** sex (*F* female, *M* male)

Duckling body condition showed high and significant within-brood repeatability (*r *= 0.56, 95% CI 0.42–0.66, *P* < 0.001), i.e., the body condition of ducklings from the same brood tends to be similar. Duckling body condition was strongly positively related to standardized residual mean egg weight (Fig. 5a) and male ducklings had a slightly lower body condition at hatching than females (Fig. 5b). No other predictors or interactions were retained in the final model (Table 3).

**Table 3:** Final linear mixed effect model (in bold) and model selection of variables and interactions explaining duckling residual body weight (body condition)

Dependent variable	Predictor variables	Estimate ±SE	*t*	*P*
Duckling body condition	Sex (male)	**− 0.24 ± 0.12**	**− 2.02**	**0.046**
	Standardized residual egg weight	**0.54 ± 0.10**	**5.70**	**< 0.001**
	Maternal plasma CORT (ng/ml)	0.0083 ± 0.0076	1.08	0.28
	Clutch size	**− **0.11 ± 0.090	**− **1.18	0.24
	Hatch date	0.026 ± 0.026	1.00	0.32
	Maternal body condition	**− **0.080 ± 0.13	**− **0.60	0.55
	Breeding experience (years)	0.019 ± 0.028	0.66	0.51
	Time to maternal blood sample (s)	0.0027 ± 0.0056	0.49	0.63
	Growth (tarsus length incubation duration^−1^)	0.34 ± 0.93	0.37	0.71
	Sex × growth	1.30 ± 1.17	1.11	0.27
	Sex × breeding experience	0.021 ± 0.034	0.61	0.54
	Maternal RTL	0.031 ± 0.10	0.31	0.76
	Log(duckling fCORT) (pg/mm)	**− **0.014 ± 0.050	**− **0.28	0.78
	Sex × log(duckling fCORT)	0.042 ± 0.094	0.44	0.66
	Sex × maternal RTL	**− **0.029 ± 0.11	**− **0.25	0.80
	Sex × maternal body condition	**− **0.058 ± 0.20	**− **0.29	0.77
	Sex × maternal plasma CORT	**− **0.00030 ± 0.012	**− **0.25	0.81

Given are the parameter estimates, standard errors (SE), *t* values and the *P* values; the model included maternal ID as a random effect (*N* = 181 ducklings and 58 females)

## Discussion

The extent to which adult sexual dimorphism is shaped by the early-life maternal environment is poorly known. Here, we showed that adult eiders, exhibiting marked sexual differences in plumage characteristics (e.g., color) and in the contribution to parental care, exhibit sex-dependent associations between CORT exposure, RTL and growth already prior to hatching. Below, we aim to bring these findings together and explore their implications.

### Offspring fCORT

Because eider ducklings are size-monomorphic at hatching (Lehikoinen et al. 2008), the sex-specific relationship between growth and fCORT is unlikely due to any qualitative sexual differences in growth trajectories per se. Higher fCORT was associated with faster growth of male embryos. This finding challenges the views from laboratory experiments that exposure to glucocorticoids during development retards growth (e.g., Spencer et al. 2003), particularly in males (e.g., Cote et al. 2006; Hayward et al. 2006). However, CORT may also accelerate early growth, thereby enhancing antipredator and locomotor functions, which may aid survival (‘CORT-activity hypothesis’: Breuner and Hahn 2003; Rivers et al. 2012). Increasing evidence also suggests that female birds may in fact be more susceptible to early-life CORT than males (Verhulst et al. 2006; Schmidt et al. 2012; Gil et al. 2019). These sexual differences may reflect the fact that CORT and testosterone levels in eggs are typically positively correlated (Ketterson et al. 1991), and testosterone may disproportionately retard the growth of female embryos (e.g., Henry and Burke 1999).

Intriguingly, we found that maternal baseline plasma CORT levels were inversely related to offspring fCORT levels (Fig. 2a). This result may indicate that offspring steroid levels are not simply a byproduct of maternal steroid levels, through passive delivery to the embryo (‘passive model’; Moore and Johnston 2008). Furthermore, our finding should not be considered unusual: similar inverse relationships between maternal plasma CORT levels and CORT levels in eggs have also been reported before (e.g., Love et al. 2008). Navara et al. (2006) proposed that the yolk may act as a reservoir for maternally derived steroids. If this is the case, mothers depositing high levels of CORT into yolks may experience a subsequent deficit of this hormone, which may lead to a negative relationship between maternal and egg levels of CORT after laying (Love et al. 2008). Such a mechanism may operate regardless of whether maternal steroid transfer is passive or actively regulated by both the mother and the embryos.

Investment in pre-laying maternal hormone deposition may depend on maternal condition: mothers in good condition may deposit less (e.g., Love et al. 2008) or more (e.g., Gasparini et al. 2007) hormones into eggs. We failed to find a significant association between maternal body condition and duckling fCORT levels (Table 1). However, a 1-year snapshot may not adequately capture the full dynamics between maternal and offspring CORT levels. Based on a multi-year analysis from our study population, elevated maternal baseline levels of CORT in blood during incubation are associated with poorer body condition of these females (Jaatinen et al. 2013). Thus, we cannot exclude indirect associations between offspring fCORT levels and maternal condition expressed through links with maternal plasma CORT. Higher maternal baseline plasma CORT was associated with lower offspring fCORT levels (Fig. 2a), which in turn were associated with shorter RTL at hatching (Fig. 4). Shorter early-life RTL has been linked with reduced fitness in other birds (e.g., Heidinger et al. 2012; Watson et al. 2015). Consequently, while we were unable to examine the fitness consequences of variation in RTL at hatching, female eiders in poor condition may be unable to avoid potential long-term physiological costs to their offspring.

Offspring fCORT levels increased with later hatching. This finding agrees with the idea of increasing environmental harshness and/or a decline in phenotypic quality of breeders with progressing season. Thus, late-nesting females show a pronounced increase in baseline plasma CORT levels with increased reproductive effort (indexed by total clutch mass) (Jaatinen et al. 2013). Offspring fCORT also increased with clutch size, which may potentially reflect a trade-off between offspring quantity and quality (e.g., Roff 1992). Females that laid larger clutches may have produced smaller or lower-quality eggs. However, whether female eiders actually face such a trade-off is unclear. Females laying larger clutches show higher survival (Yoccoz et al. 2002) and are in better body condition at hatching (Öst and Steele 2010). Furthermore, higher fCORT levels were associated with longer, rather than shorter, RTL at hatching (Fig. 4). Apart from any physiological trade-offs, per capita post-hatch duckling survival tends to increase with increasing clutch size at hatch (Öst et al. 2008b), perhaps because of the larger dilution of predation risk afforded by a larger brood (Jaatinen and Öst 2013).

### Offspring RTL

We found a significant mother–offspring correlation of RTL only between mother–daughter pairs (Fig. 3). In contrast, most of the few published studies have found that maternal telomere length in birds is more strongly related to the telomere length of sons (e.g., Becker et al. 2015). Our finding is nevertheless not unique. Belmaker (2016) found a stronger correlation of RTL in mother–daughter pairs in tree swallows (*Tachycineta bicolor*). The absence of general patterns in sex-specific telomere inheritance highlights the urgent need for further research in this area.

We found that longer maternal RTL was associated with shorter RTL of daughters. Perhaps relevant in this respect is a recent report on black-browed albatrosses (*Thalassarche melanophrys*), showing that younger parents, presumably having longer TL, produced offspring with shorter telomeres (Dupont et al. 2018). However, extreme caution needs to be exercised when attempting to draw parallels to our present study. This is because the RTL of adult female eiders shows no significant trend with age (Noreikiene et al. 2017), and female age was not associated with offspring RTL (Table 2). Clearly, there is a need for further longitudinal analysis of the covariation between maternal and offspring RTL over the lifespan of females.

We found that duckling RTL and fCORT levels at hatching were positively correlated. This result is unexpected at first glance, considering that glucocorticoids can downregulate telomerase activity (e.g., Choi et al. 2008) and thereby accelerate telomere shortening. However, mild increases in CORT levels may actually up-regulate telomerase activity (Epel et al. 2010). Second, CORT may have growth-inhibitory effects (Spencer et al. 2003). Slower development in ovo may be associated with a smaller body size and longer telomeres at hatching. Indeed, duckling tarsus length significantly decreased with increasing incubation duration in our present data (LMM (REML): *b* = − 0.17 ± 0.056 (SE), *t* = − 3.14, *P* = 0.003, *N* = 181 ducklings/58 broods). Again, however, caution is warranted. In ovo growth and fCORT levels at hatching only showed a negative association in female offspring (Fig. 1). Incubation duration may also depend on individual quality. Thus, female eiders in good body condition, having higher hatching success (Lehikoinen et al. 2010) and survival (Ekroos et al. 2012), have a longer incubation period (Seltmann et al. 2012). A good-condition female may afford the energetic strain of a longer incubation period, which may allow producing offspring with higher survival prospects (Hanssen et al. 2002). These open questions aside, the present findings suggest that higher prenatal CORT levels are not associated with shorter telomeres. Because telomere length may be positively correlated with survival early in life (e.g., Watson et al. 2015), CORT exposure during this sensitive stage of an eider’s life need not have negative fitness consequences.

### Offspring body condition

Duckling body condition was mainly explained by direct maternal energetic investment, i.e., standardized residual egg weight. Offspring body condition is likely an important proxy of subsequent survival. Thus, the survival and recruitment of female eiders is related to their relative body condition as ducklings (Christensen 1999). It is also pertinent that body condition is individually consistent between years in breeding adult female eiders (Jaatinen and Öst 2011; Ekroos et al. 2012). Furthermore, female body condition is positively correlated with clutch size, which, in turn, is positively correlated with post-hatch duckling survival (Öst et al. 2008b).

There were no connections between duckling body condition and maternal baseline plasma CORT or maternal RTL (Table 3). Baseline plasma CORT reflects a ‘snapshot’ of the activity of the CORT-releasing hypothalamic–pituitary–adrenal axis and may, therefore, not accurately portray the actual exposure to physiologically relevant endocrine signals (Fairhurst et al. 2013). The absence of relationship between maternal RTL and duckling body condition is not entirely unexpected on theoretical grounds. As suggested by Monaghan and Ozanne (2018), rates of biological aging (indexed by telomere attrition) could be largely independent of levels of maternal resources transferred to the developing embryos (‘resource-independent trade-offs’). Consequently, there may be no association between maternal RTL and offspring body reserves.

### Conclusions and future directions

This study suggests that the early developmental environment may exert heterogeneous effects on the sexes, and that prenatal CORT exposure need not be associated with shorter telomeres. However, due to the correlative nature of this study, the extent to which specific offspring phenotypes represent adaptive plasticity under natural conditions is unknown. We also encourage studies investigating the relative control that mothers and offspring have in regulating maternal glucocorticoids. Selection may favor resistance mechanisms minimizing the costs incurred by the sex facing the largest costs of maternal glucocorticoids (Sheriff et al. 2017). Individual-based, longitudinal data will be needed to assess the long-term fitness consequences of early-life exposure to maternal glucocorticoids.
